# Lessons learned from the lived experiences of people living with obesity during the first COVID-19 lockdown in the United Kingdom

**DOI:** 10.1038/s41366-025-01763-z

**Published:** 2025-04-10

**Authors:** Friedrich C. Jassil, Stuart W. Flint, Adrian Brown

**Affiliations:** 1 Centre for Obesity Research, University College London, London, UK; 2 Bariatric Centre for Weight Management and Metabolic Surgery, University College London Hospital NHS Trust, London, UK; 3 National Institute for Health Research, UCLH Biomedical Research Centre, London, UK; 4 School of Psychology, University of Leeds, Leeds, UK; 5 Scaled Insights, Nexus, University of Leeds, Leeds, UK

**Keywords:** Weight management, Epidemiology

## Abstract

**Background:**

Quantitative studies have shown that people living with obesity experienced deteriorations in mental health and health-related behaviours during the first UK’s Coronavirus Disease-2019 (COVID-19) lockdown. However, there is a lack of qualitative research exploring their lived experiences during this period.

**Methods:**

Thematic analysis of large-scale free-text survey data was conducted to understand the challenges faced by adults with obesity during the first UK’s COVID-19 lockdown.

**Results:**

Among 543 participants, 467 (86%) responded to the free text questions. The majority were female (87.8%), with a mean age of 51.6 (SD 9.9) years. Of these, 65.3% has a body mass index ≥40 kg/m^2^, and 57.7% were not enroled in weight management services. Five overarching themes and 10 sub-themes were identified with the five key themes being (1) increased fear and anxiety, (2) the impact of obesity being classified as ‘high risk’, (3) disruption in weight management services, (4) the impact on health-related behaviours, and (5) the adverse impact on mental health. Participants expressed fear of contracting COVID-19 and concerns about weight gain. UK Government messages linking obesity with severe COVID-19 complications exacerbated feelings of shame and stigma. The reduced provision of weight management services caused further health concerns, highlighting the need for digital health technologies for continued support. Participants reported changes in shopping, diet, physical activity, and sleep patterns, leading to deteriorated mental health.

**Conclusion:**

People living with obesity experienced distinct challenges during the first COVID-19 lockdown, affecting their ability to practice and maintain health-related behaviours.

## Introduction

Coronavirus Disease-2019 (COVID-19) resulted in 58,186 recorded death among adults in England during March-December 2020 [1]. In the early phase of the pandemic, obesity was immediately identified as a ‘high risk’ health status as it was associated with higher cases of hospitalisation, severe complications, and mortality rate [2–4]. This led to the Public Health England extending the Shielded Patient List to include people living with obesity [5], as well as the development and implementation of a national campaign ‘Tackling Obesity’ [6]. During the pandemic, weight management services were severely disrupted, with no access in the majority part of the country [7].

Our previous publication of the quantitative data [8] from this study shows that during the first COVID-19 lockdown, most people living with obesity experienced a deterioration in their mental health and health-related behaviours (diet, physical activity, and sleep). Specifically, 55% reported that their diet became unhealthier, 61% indicated that their physical activity had reduced, and 80% experienced worse sleep. Higher depression and lower well-being scores were found to be associated with the greatest adverse impact on health-related behaviours. These negative impacts were more pronounced among those attending weight management services pre-COVID-19 pandemic [8]. Other UK studies reported similar trends, indicating that adults with a body mass index (BMI) ≥ 35 kg/m^2^ tended to experience a reduction in their physical activity, deterioration of diet quality, and a greater frequency of overeating [9]. At a later stage of the COVID-19 pandemic, our quantitative data further revealed that some of these negative impacts such as emotional eating, worsening sleeps, and deteriorating mental health continued to persist [10].

Qualitative data is equally valuable for providing in-depth insights into how people living with obesity coped during the pandemic, offering supporting evidence for the quantitative findings. However, qualitative studies exploring the lived experience of UK adults living with obesity during the pandemic are scarce. One UK study interviewed participants from an online behavioural weight management programme to assess how COVID‐19 had impacted their weight loss journey [11]. Participants described that factors such as perception of risk, environmental and social changes, and personal well‐being influenced their motivation to lose weight. However, two qualitative studies that recruited participants from Irish hospital-based weight management clinics reported that the COVID-19 have both positive and negative impact on their health (diet and physical activity) and psychosocial well-being, in addition to increased feeling of weight stigma [12, 13]. It is important to note that these qualitative findings are limited to those actively engaged in weight management programmes during the pandemic [11–13]. Hence the findings may not be generalizable to people living with obesity who experienced cancellation or delay of their weight management support, or those who were not enroled in any form of weight management programme. To address this limitation, the present study aimed to provide in-depth insights into how people living with obesity coped during the first COVID-19 lockdown using a large, heterogeneous UK sample.

## Methods

### Study design and participants

A cross-sectional online survey design was used and hosted by University College London (UCL) Opinio. Participants were recruited across the four nations of the UK (England, Wales, Scotland, and Northern Ireland) via social media advertisements, UK professional and patient obesity organisations and the National Health Service (NHS) weight management services between the 14th May and 9th July 2020. Survey inclusion criteria were adults living with obesity (BMI ≥ 30 kg/m^2^) aged between 16 to 80 years, and/or attending Tier 2/3 or Tier 4 weight management services. Prior to completing the online survey, participants read the information sheet and provided electronic informed consent. The survey was comprised of five sections that included free-text optional questions to elicit additional information from participants (Supplementary S1). The study was conducted in accordance with the Declaration of Helsinki [14], approved by the UCL Research Ethics Committee (REC number 16191/004), and followed the Consolidated Criteria for Reporting Qualitative Research (COREQ) guidelines for qualitative research reporting [15].

### Data analysis

The free-text responses were analysed using an inductive form of thematic analysis [16]. Given the limited knowledge of the lived experiences of people living with obesity during the first COVID-19 lockdown, the aim of the current study was not to test a specific theory, but rather to take an inductive approach that identified points of salience in participants’ own accounts of their experience. Initially, two researchers (FCJ and AB) independently reviewed 20% of the free texts to familiarise themselves with the data and coded them. Using this initial framework of codes, FCJ then continued coding the remaining free texts. FCJ and AB continued to meet weekly to discuss new codes and refine them, until no more new codes were generated from the data. Next, FCJ and AB independently extracted the codes that shared similar ideas and concepts to represent broader level categories. Both FCJ and AB discussed the framework of categories and refined them through an iterative process until a consensus was reached. The reviewed categories were organised into potential themes and sub-themes. Next, the codes and themes were reviewed and refined to ensure that the themes demonstrated a valid, accurate and coherent pattern. When all themes were finalized, the names of the themes were refined to check that they provided a valid account of the data that they represent. Specific quotations were extracted to illustrate the themes and subthemes with some of the stigmatising languages in the original quotations were replaced to people-first language. FCJ and AB are dietitians with training and experience in conducting and analysing qualitative data. AB conceptualised, executed and led the survey, whereas FCJ was not involved in the wider survey.

## Results

### Demographics

Among 543 participants (Table 1), 467 (86%) provided a response to at least one of the free-text optional questions. The response rates for each of the free text question are detailed in Supplementary S2. Participants were predominantly female (87.8%), residing in England (90.4%), white (British/Irish/Other) (92.3%) with a mean age of 51.6 (SD 9.9) years and a median BMI of 37.7 (IQR, 36.0, 48.7) kg/m^2^. Of all participants, 65.3% reported a BMI ≥ 40 kg/m^2^ and 57.7% did not enrol in any form of weight management services.

**Table 1:** Baseline characteristics of the participants.

Characteristics	Mean (SD)
Age, years	51.6 (9.9)
Gender, *n* (%)	
Male	66 (12.2)
Female	477 (87.8)
Ethnicity, *n* (%)	
White - British, Irish, other	501 (92.3)
Asian/Asian British	8 (1.5)
Black/Black British	9 (1.7)
Chinese/Chinese British	4 (0.7)
Middle Eastern/Middle Eastern British	2 (0.4)
Mixed race - White and Black/Black British	5 (0.9)
Mixed race - other	8 (1.5)
Other ethnic groups	3 (0.6)
Prefer not to say	3 (0.6)
Weight (kg), median (IQR)	103.4 (97.0, 137.0)
BMI (kg/m^2^), median (IQR)	37.7 (36.1, 48.7)
Country of Residence, *n* (%)	
England	453 (90.4)
Wales	14 (2.8)
Scotland	23 (4.6)
Northern Ireland	11 (2.2)
Living with obesity, *n* = 540 (*n*, [%])	503 (93.1)
Body Mass Index ≥ 40 kg/m^2^, *n* = 539 (*n*, [%])	352 (65.3)
Bariatric surgery, *n* = 539 (*n*, [%])	83 (15.3)
Surgery type, *n* (%)	
Roux-en-Y Gastric Bypass	28 (34.6)
Sleeve Gastrectomy	37 (45.7)
Laparoscopic Adjustable Gastric Banding	11 (13.6)
Biliopancreatic Diversion	1 (1.2)
Duodenal Switch	1 (1.2)
Mini Gastric Bypass/ One Anastomosis Gastric Bypass	2 (2.5)
Other	1 (1.2)
At the time of COVID-19 outbreak, were you attending? *n* = 504, (*n*, [%])	
Tier 2—GP and commercial weight management services	65 (12.9)
Tier 3—Specialist weight management services	54 (10.7)
Tier 4—1st appointment bariatric services	26 (5.2)
Tier 4—Awaiting surgery/pre-op diet	31 (6.2)
Tier 4—Post-bariatric surgery	37 (7.3)
No current programme	291 (57.7)

*n*, number, *COVID-19* Coronavirus Disease-2019, *GP* General Practitioner, *IQR* interquartile range, *SD* standard deviation.

### Themes

**Fig. 1:**
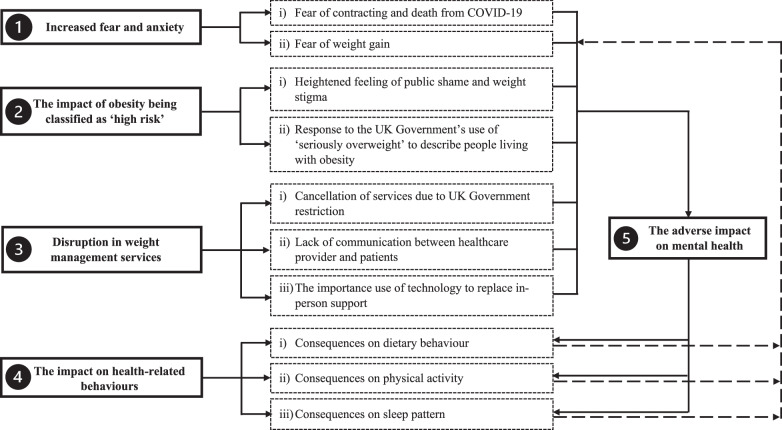
A thematic map illustrating the themes and subthemes (including their interactions) that representing the lived experiences of people living with obesity during the first COVID-19 lockdown. COVID-19, Coronavirus Disease-2019.

Five overarching themes and 10 sub-themes being generated from the data and their interactions are shown in Fig. 1. These five themes were (1) increased fear and anxiety, (2) the impact of obesity being classified as ‘high risk’, (3) disruption in weight management services, (4) the impact on health-related behaviours, and (5) the adverse impact on mental health. Additional examples of quotations for themes and subthemes are also presented in Supplementary S3.

### Theme 1: Increased fear and anxiety

Most participants described how the first COVID-19 lockdown had increased their fear and anxiety and expressed concerns about the negative impact it caused on their body weight.

### Sub-theme (i) Fear of contracting and death from COVID-19

Most participants expressed strong fear of the risk contracting the severe form of COVID-19 with lower chance of survival, poor recovery with long-term damage, or even death due to living with obesity and its underlying health conditions, *“If I catch it, I am highly likely to die due to weight”* (Participant 418 [P418]). They also expressed worry that they would receive lower priority for medical treatment if they were hospitalised during COVID-19 due to their body weight. The prospect of leaving their loved ones behind in the event of not surviving the infection weighed heavily on their minds, *“I am concerned that I might not receive optimal treatment if taken severely ill, which is a problem as I am family breadwinner”* (P28). They also feared transmitting the virus to their loved ones who may either become seriously ill or die; for instance, *“catching it, passing on to family members and [them] dying”* (P411).

### Sub-theme (ii) Fear of weight gain

A consistent and increasing concern reported by participants was weight gain due to uncontrolled eating habits and reduced physical active (detailed in Theme 4), which they felt further increased their vulnerability to COVID-19 infection. For example, *“..that I will gain more weight or contract COVID-19 and become very unwell*” (P455). For some participants, they feared that weight gain may worsen their current health conditions and delay treatment they were due to or on the waiting list to receive. For example, this participant comments on their concerns relating to their pre-COVID-19 planned bariatric surgery, *“I lost the 5% weight required for surgery but unfortunately, due to the pandemic, the surgery has been put on hold which is very difficult for me. I cannot control my weight. It has been going up and down”* (P403).

### Theme 2: The impact of obesity being classified as ‘high risk’

The UK Government’s announcement of the link between obesity and COVID-19 complications intensified feelings of shame and stigma among participants. Concerns were also raised over the suitability of using the term ‘seriously overweight’ in the Shielded Patient List.

### Sub-theme (i) Heightened feeling of public shame and weight stigma

Participants perceived that the UK Government’s announcement had increased their feelings of self-blame, *“Feeling blamed for being high risk, feeling less valued, feeling like a problem”* (P291). This led them to consider themselves as a burden to their family and also healthcare services, *“Guilty that if infected, I would take up resources if hospitalised; worries others and health professionals would blame me”* (P26). The announcement was also perceived as targeting people living with obesity, which was felt to increase societal weight stigma, as evidenced by comments posted on social media as well as the public reaction, *“I have always felt stigmatised, however this feeling has increased due to jokes, memes circulating at this time… It’s led to a backlash from the general public and the old adages of eat less, move more, name calling etc.”* (P295). Hence, some participants debated that the UK Government should have provided support for people living with obesity rather than just an announcement. This lack of support appeared to lead to feelings of hopelessness and helplessness, with this participant reporting they felt *“Awful and attacked yet without any kind of helpful constructive support other than lose weight which is easier said than done”* (P339). Oppositely, there were a small number of participants reported feeling that the announcement made them feel more cautious to comply with the preventive measures against COVID-19 and motivated them to try to lose weight, *“It has made me want to lose weight”* (P485).

### Sub-theme (ii) Response to the UK Government’s use of ‘seriously overweight’ to describe people living with obesity

The UK Government’s use of the term ‘seriously overweight’ in the Shielded Patient List sparked debate and scrutiny by the majority of participants. They felt that the term was too ambiguous, difficult to quantify, did not take into account body composition, and was open to interpretation, with this participant expressing, *“Different people may have a different idea of being seriously overweight. It’s a vague statement”* (P189). They also suggested that using BMI or obesity categories was a much better way of describing those at ‘high risk’, *“Perhaps they could use the obesity class 1, class 2 categories”* (P20). Furthermore, they believed that the term ‘seriously overweight’ was negative language and sounded “labelling”, “offensive”, “rude”, “degrading”, “derogatory”, and “demoralising”, with this participant saying, *“It’s derogatory in a way and could make people feel stigmatised”* (P179).

In contrast, those who found the term acceptable argued that the term ‘seriously overweight’ was a more appropriate choice of word compared to more stigmatising words like “obese”, “morbidly obese”, or “grossly obese”. Here, this participant explains how they preferred this term to other more stigmatising language, *“I don’t think there is any nice way to describe people with overweight, I think I prefer to be called seriously overweight than morbidly obese”* (P327). Given that not everyone knew their BMI, the term was considered the best way to describe people living with excess body weight in general. It was felt to convey a clear meaning, provided an appropriate description, and importantly, it did not promote the participant having feelings of self-blame, with this participant explaining, *“I think this is fair language and is clear - enough to know who it means without apportioning blame”* (P30).

### Theme 3: Disruption in weight management services

The UK Government’s advice to suspend face-to-face weight management services led to the cancellation or delay of these services, causing frustration and health concerns among participants. Additionally, the lack of communication with healthcare providers highlighted the importance of using technology to enable continued contact, support, and care during the COVID-19 pandemic.

### Sub-theme (i) Cancellation of services due to UK Government restriction

Most participants understood that Tier 2 and 3 weight management services (community and specialist obesity services) were cancelled or delayed to prioritise the NHS’s pandemic response, though some felt disappointed and frustrated by it, this participant expressed feeling, *“Sad and frustrated. However, understand the reasons why. I just feel that it’s been sidelined and forgotten”* (P468). Meanwhile, those in Tier 4 weight management services (bariatric surgery) expressed concerns that the delay in receiving bariatric surgery would lead to further deterioration of their health, *“I have been waiting for months and my weight is affecting my joints so I can’t exercise at home, affecting how well I feel”* (P524). Participants also reported that bookings for consultations, B12 injections, and blood tests became very difficult. Without clinical support, their weight management journeys became more challenging, and they felt “sad”, “helpless”, “frustrated”, and “worried”. Some participants mentioned losing motivation, becoming less focused, losing track, and feeling out of control. Overeating, reverting back to old poor eating habits, weight gain, and deteriorating health status were commonly cited as negative outcomes. Here this participant explained how the cancellation of services had impacted their eating, fear of weight gain, and worries about not getting bariatric surgery, *“I have become obsessed with food again as being at home all the time on my own is very hard. I’m fearful that as I’ve put weight on, I will not be approved for surgery and that scares me!”* (P257).

### Sub-theme (ii) Lack of communication between healthcare provider and patients

Some participants reported that all individual and group sessions had been halted, leaving them without any communication with their clinical teams. Whilst a minority mentioned ongoing contact, they noted a significant reduction in communication frequency. Consequently, some participants felt abandoned due to the loss of contact, leading to uncertainty about their status in the weight management services pathway, *“Obviously no physical appointments, but there haven’t been any general letters sent to all patients regardless of which tier or pathway they are on, saying what is going on and plans for the way forward”* (P232).

### Sub-theme (iii) The importance use of technology to replace in-person support

Participants who received continuous care reported that all their face-to-face appointments had been converted to remote phone and video calls, with group sessions conducted via video call platforms such as Zoom. Additionally, the clinical team utilised text messages and email to communicate with them, “*Obviously there is no more face to face, every communication is now via messages, emails, phone and Zoom”* (P405). Some participants also mentioned finding updates about their weight management services on social media platforms shared by their peers. Whereas others suggested that more available online resources from their clinical teams would have been beneficial to help them manage their weight during the stressful lockdown period. Here, this participant emphasised the need for more online resources, *“I wanted to be able to access information online… there is no website or programme to follow, everything is about signposting to other websites”* (P469).

### Theme 4: The impact on health-related behaviours

Participants reported that COVID-19 pandemic led to changes in dietary behaviours, physical activity levels, and sleep patterns. The majority reported that the first COVID-19 lockdown has a negative impact, though some expressed that this time had positively impacted their health-related behaviours.

### Sub-theme (i) Consequences on dietary behaviour

Most participants explained that they had resorted to online shopping and deliveries, as well as the click and collect services. They also reported that the lockdown meant that only one person from the household (either “husband” or “partner”) did the shopping, whilst others said that they either helped other family members or received helps from other to do shopping on their behalf, “*Don’t go to the shop. Do it online or send someone else to do it for me”* (P200). Physical shopping became an essential activity rather than recreational with participants reporting they shopped significantly less frequently, that it required more planning in advance and became quicker due to less browsing, but then more time consuming due to the longer queue to purchase their shopping. Some participants reported buying more junk foods and less fresh produce, with this participant reporting, *“Shop less often, buying more unhealthy foods and less vegetables. Buying quick and easy meals as have no motivation to cook”* (P146).

These changes in shopping behaviour and access to foods appeared to impact on their food choices. Participants reported eating less fruits and vegetables with generally less protein, but a greater intake of carbohydrate, *“I can’t get enough fresh food and I’m eating more carbs”* (P149). The majority of participants also reported an increased frequency of overeating, comfort eating, and emotional eating as coping strategies to deal with feelings of boredom, loneliness, and depression. This subsequently led to greater consumption of high calorie foods and drinks including alcohol and more takeaways, *“Eating more unhealthy foods including snacks like biscuits. Not eating as many vegetables. Buying more takeaways”* (P145).

In contrast, for some participants, the lockdown had a positive impact, providing them with more time to plan and prepare healthy meals for their families, leading to fewer takeaways, *“Not buying as many takeaways, home cooking more and buying processed foods less”* (P87). For some, the lockdown motivated them to start losing weight due to concerns about the link between obesity and severe COVID-19 complications, with participants reporting practicing various forms of dieting, such as “low carbohydrate diet”, “calorie counting”, “meal replacement diet”, “Mediterranean diet” and “intermittent fasting”, “*Lost 18 lbs as concerned about obesity and COVID. Lost via intermittent fasting”* (P21).

### Sub-theme (ii) Consequences on physical activity

The COVID-19 lockdown resulted in many participants being unable to maintain their previous levels of physical activity. This was caused by reductions in their activities of daily living, such as not taking their children to school, not commuting to work due to remote working, being furloughed or job loss, and limitations on outdoor activities, *“I have not been out and about as I normally would do. Currently no school runs, and I am not working either”* (P328). The closures of fitness centres and gyms further limited their opportunities for structured exercise, making it harder to stay active. This participant explains about the closure of their gym and their feeling of loss and impact on their health, *“No longer able to swim at the gym. Swimming has been a great loss, put more weight on and muscles are weaker”* (P240). Furthermore, loss of motivation, impaired physical function, deteriorating health conditions, fearful of going out, and limited time outdoor were among the barriers faced by participants to engage in physical activity. This was even worse among those who reported shielding, as they were confined to their homes, often with limited access to outdoor spaces for exercise, *“I am shielding so not doing any day to day walking”* (P27).

Conversely, some participants found that the COVID-19 lockdown allowed them to spend greater time on physical activity than pre-pandemic. Many reported taking up activities such as gardening, walking, and cycling, with popular programmes such as the Couch to 5k app and online exercise classes, *“Have got less incidental exercise (such as shopping trips, work) but bursts of more intense exercise, e.g. jogging, exercise bike, Joe Wicks [free online workouts known as ‘PE With Joe’]”* (P30). Additionally, participants reported variations in motivation, with some initially engaging in more physical activity but subsequently this tapering off, while others experienced the opposite, *“When first in lockdown I walked every day. Then I went from that to doing nothing for weeks. And now I’m back to trying to do some exercise. Very inconsistent”* (P443).

### Sub-theme (iii) Consequences on sleep pattern

Most participants reported their sleep pattern was negatively impacted during the first COVID-19 lockdown. This was attributed to several factors, including disruptions to daily routines, increased sedentary behaviour resulting from working from home and reduced physical activity, and a general sense of anxiety and worry, *“I suppose stress and anxiety have interrupted sleeping patterns. Finding it difficult to get to sleep and stay asleep”* (P162). Many participants struggled with falling asleep, experienced restless nights, and found themselves waking frequently throughout the night, often unable to return to sleep, *“Wake up three or four times in night, don’t get to sleep till 3am, then don’t get up till 11am”* (P253). Feelings of exhaustion and fatigue were commonly reported, leading some to resort to daytime napping to cope with boredom, *“I am unable to sleep until very late at night because of pain which means I end up sleeping into the afternoon. This is also partly due to my mood”* (P25).

Variability in sleep quality was also mentioned, with some individuals experiencing periods of restful sleep followed by bouts of difficulty sleeping, accompanied by vivid and disturbing dreams. These issues appeared to be linked to feelings of isolation from family, fear of contracting COVID-19, and pre-existing medical conditions such as physical pain*, “I used to get to sleep really well and sleep all night, but now I am having bad dreams and am very restless. This could be due to other aspects of my life such as not seeing family”* (P35). On the other hand, a minority of participants reported improved sleep due to working from home, which eliminated their daily commute. They experienced reduced stress from not being at work and benefited from the opportunity to sleep in without early morning commitments such as school runs, “*Sleeping well most nights, as no stress from work colleagues, and no rushing to work in mornings so can rest longer if needed”* (P15).

### Theme 5: The adverse impact on mental health

Most participants described how the COVID-19 pandemic and the measures undertaken by the UK Government disrupted their routines and caused them to be socially isolated leading to feelings of “sadness”, “confusion”, and “loneliness”. Here a participant recalls how she felt during lockdown, *“Lonely despite living with two of my children, scared to the point of panic attacks… and lethargic”* (P156). They also experienced being unmotivated and unproductive, which significantly affected their mental health, for example this participant reported feeling *“Frustrated. Mentally I want to do something about it, but while I know what I need to do, I just can’t get motivated”* (P367). Some participants felt worried, frightened, concerned, and depressed due to the increased risk of COVID-19 infection and the poor survival associated with living with a higher BMI, especially following the UK Government’s announcement for shielding, with this participants expressed her concerns about COVID-19, *“Scared, helpless and definitely has impacted my mental health, at times, I feel certain if I was to get COVID-19 I wouldn’t survive it”* (P280). Additionally, stigmatising media portrayal about people living with obesity alongside the negative public response, and those ignoring the UK Government rules further worsened their mental health, *“Increased my depression and anxiety, made me very angry at the nonchalant attitude of others”* (P395).

The cancellation of weight management services and the lack of communication from their healthcare providers also left participants feeling disappointed, depressed, and anxious. Without clinical support, their weight loss journey became difficult, leading to further feelings of sadness, helplessness, frustration, and worry, *“Poor communication and lack of support for my mental health and my weight has made me feel a lot worse than before the outbreak”* (P472). As a result, some participants reported turning to emotional eating, losing motivation for physical activity, and experiencing increased sleep problems, all of which exacerbated weight gain and deteriorated their overall health, “*Depression increased due to social isolation, this led to not being able to self-care, including food preparation and maintaining a regular diet due to poor mental health. I have put 2½ stones back on after losing 3 stones up until lockdown”* (P395). Therefore, some participants suggested that mental health support would have been beneficial, “*Providing people living with obesity with online psychological support during this time. They tell us to lose weight and that its important, but they do nothing to help us”* (P25).

## Discussion

Our qualitative data from people living with obesity in the UK revealed an increased fear of contracting and dying from COVID-19, compounded by concerns about weight gain due to lockdown, which they felt may have further elevated their risk of COVID-19 complications. UK Government and public discussions linking obesity with severe COVID-19 complications appeared to intensified feelings of shame and weight stigma. Despite the recognised risks between obesity and COVID-19 complications, disruption in the weight management services continue to persist, leading to frustration and increased health concerns among participants. Hence, they highlighted the importance of leveraging digital technology for service delivery. The first COVID-19 lockdown also impacted various aspects of their health-related behaviours, including shopping, diet, physical activity levels, and sleep patterns, either for better or worse. Together, these negative impacts led to a self-reported deterioration in mental health.

Despite the heightened fear of severe outcomes from COVID-19 infection among people living with obesity and the UK Government’s campaign to promote weight loss [6], many participants reported experiencing weight gain. A previous study revealed that being well-informed about obesity and COVID-19 risk did not always translate into increased motivation to lose weight [17]. Furthermore, public health messages that underplaying the complexity of obesity and using language that promotes weight stigma and discrimination are ineffective in promoting health-related behaviours [18] and, if anything, caused them to worsen. This was indeed the case for the UK policy response to obesity during the first COVID-19 lockdown, which was heavily criticised for promoting a culture of blame and shame on people living with obesity, inadvertently perpetuating more harm than good [19, 20]. A systematic review and meta-analysis showed that weight bias internalisation resulting from weight stigma and discrimination was associated with poorer dietary adherence, alongside reduced motivation and self-efficacy to practice health-promoting behaviours [21]. Therefore, it is not surprising that experiencing weight stigma and discrimination may have in part been a driver for people living with obesity gaining weight over the lockdown [22].

With participants reporting frustration about the lack of access and support from weight management services, this suggests that the UK Government’s advice to ‘lose weight’ was not sufficiently supported with necessary actions and resources. This is despite the publication of a new obesity strategy during the COVID-19 pandemic, which again implies that the suggested strategies were only a surface-level solution [23] and insufficient to help people living with obesity to lose weight. As shown by data from the COMS-UK study, weight management services were severely impacted during the pandemic, with 97.8% of elective bariatric surgeries cancelled, 67.3% of units halting multidisciplinary meetings, and 69.6% reporting reduced clinics [7]. This highlighted the necessity of adopting alternative methods of service delivery including digital technology. Since the COVID-19 pandemic, a National Institute for Health and Care Excellence (NICE) early value assessment on digital technologies for delivering multidisciplinary obesity services has been undertaken, resulting in nine digital services being approved to deliver digital weight management programmes within the NHS [24]. This aligns with the recently updated NICE obesity guidance aimed at improving access to multidisciplinary weight management services, including for people who do not have access to a specialist weight-management service in their area [24]. However, digital poverty and illiteracy experienced by people from socioeconomically disadvantaged group, may subsequently impact the uptake of such programmes [25], therefore hybrid approaches are recommended [26, 27].

Data from this study expand on our previous quantitative findings [8] that the first COVID-19 lockdown negatively impacted health-related behaviours of most participants, though this was not experienced by everyone. The unfavourable changes in eating habits reported in the present study are similar to those observed in the general population [28, 29]. The reduced consumption of fresh food was predominantly due to reduced physical access during the first COVID-19 lockdown [30], which at a later stage of the pandemic was primarily attributed to food insecurity as shown by our data [10]. Whereas increased intake of high-calorie food, alcohol, and frequency of grazing were associated with maladaptive coping mechanisms, such as comfort eating to alleviate emotional distress during the lockdown [28]. Our data also corroborates the previous findings that demonstrated a positive link between poor diet quality and higher emotional eating among UK adults with a higher BMI [9, 31].

Previous UK studies reported that during the COVID-19 pandemic, higher BMI was associated with lower levels of physical activity [9], with increases in sedentary lifestyle were linked to the changes in daily routines and the closure of gyms and leisure facilities [32]. Our qualitative data further expands on this, revealing that a perceived loss of motivation, impaired physical function, concerns about contracting and dying from COVID-19, and deteriorating health conditions were additional barriers faced by people living with obesity, which may help to partially explain the abovementioned association. It is worth noting that a subset of participants in this study reported that they either continued practicing healthy dietary behaviours and physical activity or that the first COVID-19 lockdown afforded them the time and space to make positive lifestyle changes. This can be seen from the improved eating habits reported by some participants, such as spending more time planning and cooking meals and reducing takeaway foods. These positive changes have also been observed in the general population [28, 29]. A perceived lack of time related to work, education, and family commitments is indeed one of the commonly reported barriers to practicing healthy dietary habit and performing physical activity among people living with obesity [33, 34]. With restrictions now ceased and people returning to work following the COVID-19 pandemic, this may have posed a challenge for people to continue these positive changes to their behaviour. Therefore, it is important that healthcare professional appreciate the impact of work, commuting, and time play on changing health-related behaviours.

Our qualitative data demonstrated variable changes in sleep quantity and quality during the first COVID-19 lockdown relative to pre-pandemic, though appeared to show that most participants experienced a deterioration in this time. This corroborates our quantitative findings and that of others [8, 35]. Factors such as increased fear, stress, anxiety, a sedentary lifestyle, reduced early morning commitments, and decreased work-related stress have been shown to influence sleep patterns during the COVID-19 pandemic [36]. Our qualitative data further revealed that other issues like fatigue, physical pain, pre-existing medical conditions, and social isolation also play significant roles in disrupting sleep patterns among people living with obesity. With insufficient sleep being linked to unfavourable appetite responses, leading to increased calorie consumption [37], and poorer sleep quality was associated with greater weight gained during the COVID-19 pandemic [35], this may in part explain the reasons participants reported gaining weight over the first COVID-19 lockdown.

Deterioration in mental health was observed among the UK general population during the first COVID-19 lockdown [38], with our quantitative data indicating this was also the case for people living with obesity [8]. The present qualitative data expands on this understanding, showing that the negative impact on mental health was attributed to a myriad of factors. These include an increased fear of death from COVID-19, intensified by the UK Government and public discussions linking obesity with severe COVID-19 complications which subsequently lead to heightened feelings of public shame and weight stigma. This was further exacerbated by reduced access and support from weight management services. We found that this deterioration in mental health led to emotional eating, poor motivation to performing physical activity, and disrupted sleep patterns. The Fenland COVID-19 app study demonstrated that for every incremental increase in depression severity score predicted a weight gain of 0.045 kg one month later [39], which may also help to explain why participants were self-reported they had gain weight.

A strength of this study is its large sample size and data-driven approach utilising thematic analysis. This study helps to expand on current understanding regarding the impact of the first COVID-19 lockdown which has mainly focused on quantitative data analysis, by offering greater granularity in understanding the challenges faced by people living with obesity during this time. This study however has several limitations which need to be appreciated when interpreting this data. Despite significant attempts, there was a smaller representation of males and people from ethnic minority groups. This potentially limits the generalisability of the study findings. This study was cross-sectional, and therefore only offers insights about the lived experience of the first COVID-19 lockdown period. Thus, inferences of how people living with obesity were impacted throughout the COVID-19 pandemic cannot be drawn. Additionally, incomplete free-text responses of the survey from some participants may have introduced bias, affecting the interpretation of the study results. Finally, the online recruitment method may have excluded individuals experiencing digital poverty and thus, the lived experience of people living with obesity from the most disadvantaged groups may not have been captured.

### Implications of findings for the future

The lessons learned from this qualitative study have several implications for a wide range of groups, including people living with obesity, policymakers, healthcare professionals, and the general public. These include the need to reframe health messages, avoid stigmatising language, leverage digital health technology, and improve access to mental health services (Table 2).

**Table 2:** Recommendations to support people living with obesity.

**Reframe health messages**	Policymakers should reframe health messages from using fear and scare tactics and move towards approaches like hope messaging, which has been proven to foster positive motivation for behavioural change [29, 40].
**Avoid stigmatising language**	It is crucial to avoid stigmatising language when referring to people living with obesity to mitigate public shame and weight stigma.
**Leverage digital health technology**	Adoption of digital health technologies is essential to ensure continuous, scalable, individual-level weight management services.
**Improve access to mental health services**	Mental health support should be provided alongside weight management services to address the holistic needs of people living with obesity, especially in times of unprecedented crises such as a worldwide pandemic.

## Conclusions

The COVID-19 pandemic and the ensuing first UK lockdown negatively impacted the lives of people living with obesity, as they faced distinct challenges in practicing and maintaining health-related behaviours. The severe disruption in weight management services and the lack of support during this period underscore the need for UK healthcare systems to be better prepared and equipped to support people living with obesity in the event of future pandemics.

## Supplementary information

Supplemental Material

## Data Availability

The dataset generated during and/or analysed during the current study are available from the corresponding author on reasonable request.
