# Supplementary material for: Lessons learned from the lived experiences of people living with obesity during the first COVID-19 lockdown in the United Kingdom
Source: Int J Obes (Lond). 2025 Apr 10;49(6):1173–80. doi: 10.1038/s41366-025-01763-z (PMC12158763; doi:10.1038/s41366-025-01763-z)
Supplement: Supplementary file 1 — Supplemental Material [file 41366_2025_1763_MOESM1_ESM.docx]

**Lessons learned from the lived experiences of people living with obesity during the first COVID-19 lockdown in the United Kingdom.**

Friedrich C. Jassil^1-3^, Stuart W. Flint^4,5^, Adrian Brown^1-3*^

1. Centre for Obesity Research, University College London, London, UK
2. Bariatric Centre for Weight Management and Metabolic Surgery, University College London Hospital NHS Trust, London, UK
3. National Institute for Health Research, UCLH Biomedical Research Centre, London, UK
4. School of Psychology, University of Leeds, Leeds, UK
5. Scaled Insights, Nexus, University of Leeds, Leeds, UK

**Supplementary S1:** Survey questions and structure

**Block 1: Demographics**

1. Please state your age
2. Gender - male, female, other, prefer not to say
3. Ethnicity – standard answers
4. Please provide the first half of your postcode
5. Height
6. Weight
7. Do you identify as being a person living with obesity? Yes No
8. Do you have a body mass index (BMI) of 40 or above? Yes No
9. Have you had bariatric surgery? Yes No

- If yes which surgery have you had? Roux-en-Y gastric bypass; Sleeve gastrectomy; Gastric Band; Biliopancreatic diversion (BPD); Duodenal switch (DS), Mini gastric bypass / One anastomosis gastric bypass (MGB/OAGB); other (please specify in the text box)
- What year did you have your operation?
- If no, at the time of COVID-19 outbreak were you:
  - attending a local weight management service (Tier 2) e.g. GP practice, Weight Watcher Referral
  - attending a specialist weight management service (Tier 3) with or without a view for surgery referral
  - awaiting to be seen by for first appointment with bariatric surgery services
  - awaiting bariatric surgery with a planned date for your bariatric surgery and/or started your liver shrinkage/pre-op diet in preparation for have surgery
  - Had bariatric surgery and were currently being followed up
  - Currently not part of a formal weight management programme incl. being discharged from services above

1. Have you actively been attempting to lose weight since the COVID-19 outbreak? Yes No
2. Please provide a unique identifier that can be used in follow up surveys e.g. first two letters of postcode and last two numbers of date of birth.

**Block 2: Awareness, thoughts and actions relating to COVID-19**

1. Do you have any of the other health conditions below identified by the UK Government as having an increased risk of severe illness from COVID-19?

- Aged 70 or older (regardless of medical conditions)
- Under 70 with an underlying health condition listed below (i.e. anyone instructed to get a flu jab as an adult each year on medical grounds):
  - chronic (long-term) respiratory diseases, such as [asthma](https://www.nhs.uk/conditions/asthma/), [chronic obstructive pulmonary disease (COPD)](https://www.nhs.uk/conditions/chronic-obstructive-pulmonary-disease-copd/), emphysema or [bronchitis](https://www.nhs.uk/conditions/bronchitis/)
  - chronic heart disease, such as [heart failure](https://www.nhs.uk/conditions/heart-failure/)
  - [chronic kidney disease](https://www.nhs.uk/conditions/kidney-disease/)
  - chronic liver disease, such as [hepatitis](https://www.nhs.uk/conditions/hepatitis/)
  - chronic neurological conditions, such as [Parkinson’s disease](https://www.nhs.uk/conditions/parkinsons-disease/), [motor neurone disease](https://www.nhs.uk/conditions/motor-neurone-disease/), [multiple sclerosis (MS)](https://www.nhs.uk/conditions/multiple-sclerosis/), a learning disability or cerebral palsy
  - [diabetes](https://www.nhs.uk/conditions/diabetes/)
  - problems with your spleen – for example, [sickle cell](https://www.nhs.uk/conditions/sickle-cell-disease/) disease or if you have had your spleen removed
  - a weakened immune system as the result of conditions such as [HIV and AIDS](https://www.nhs.uk/conditions/hiv-and-aids/), or medicines such as [steroid tablets](https://www.nhs.uk/conditions/steroids/) or [chemotherapy](https://www.nhs.uk/conditions/chemotherapy/)
- Pregnant
- I have been diagnosed with another short or long-term health condition not listed above e.g. mental health, obstructive sleep apnoea, (please specify in the text box)
- I am not in any other high-risk group or have another health condition

1. You have indicated that you are another one of the ‘at risk’ groups, please can you specify what health condition you have and if relevant what stage e.g. type 2 diabetes, hepatitis B, obstructive sleep apnoea
2. Have you experienced COVID-19 symptoms? Yes and I was diagnosed with COVID-19; Yes but I have not been diagnosed with COVID-19; No; I don’t know what the symptoms are or only know some
3. Which of the symptoms of COVID-19 have you experienced? (Select all that are relevant)
   - Persistent cough
   - Feeling confused
   - Loss of appetite
   - Loss of smell
   - Loss of taste
   - Tightness in chest
   - Diarrhoea
   - Fatigue
   - Shortness of breath (e.g. hoarse voice)
   - Fever (temperature over 37.8°C)
   - Sore throat
   - Other (please specify in the text box)
4. As you had symptoms have you officially been diagnosed with COVID-19? Yes, I have been diagnosed officially by testing; No I haven’t been tested but COVID symptoms
5. What is your biggest concern during the COVID-19 outbreak? (please specify in the text box)
6. Have you taken any of the actions below in response to the coronavirus (COVDI-19) outbreak? (select all that apply)

- Socially distanced ensuring that 2 metres from others (steps you can take to reduce social interaction between people e.g. lockdown)
- Self-isolation (Not leaving your home if you have symptoms of COVID-19 or live with someone who does)
- Shielding due to being defined as 'extremely vulnerable’ (minimising interaction between those who are extremely vulnerable and others)
- Worn protective apparel (e.g. gloves, masks, etc)
- Used online shopping or food delivery
- Other (please specify in the text box)

1. Do you believe that you are at higher risk of infection from COVID-19 due to your body mass index being over 40kg/m^2^ ? Yes, No
2. You identified that your BMI is greater than 30kg/m^2^ but less than 40kg/m^2^ which is not on the UK Governments criteria for higher risk of severe illness, do you still believe you are still at higher risk? Yes, No
   - You identified that your living with obesity but your BMI was less that 40kg/m^2^ which is not on the UK Governments criteria for higher risk of severe illness, do you still believe you are at higher risk of severe illness? Yes, No
3. The UK government has identified people who have a BMI 40kg/m^2^ or over as being at a higher risk of severe illness from COVID-19. Please describe how this made you feel? (please specify in the text box)
4. How has following the UK Government decision for social distancing, lockdown, self-isolation and shielding made you feel? (please identify 3-5 key words)

**Block 3: Service provision**

Individual questions for each stage of the pathway

**Tier 2**

1. Has COVID-19 outbreak resulted in your attendance to Tier 2 Weight Management (e.g. locally run slimming world) appointments being cancelled and/or delayed?
2. How has having your appointments cancelled and/or delayed made you feel?

**Tier 3**

1. Has COVID-19 outbreak resulted in your attendance to Tier 3 Specialist Weight Management appointments being cancelled and/or delayed?
2. How has having your appointments cancelled and/or delayed made you feel?

**Awaiting to have your initial appointment with the bariatric service following referral**

1. Has COVID-19 outbreak resulted in your initial appointment with the bariatric surgery team being cancelled and/or delayed?
2. How has having your initial appointment with the bariatric surgery team to discuss surgery cancelled and/or delayed made your feel?

**Had a date for your bariatric surgery and/or started your pre-operation diet in preparation for surgery**

1. Has COVID-19 outbreak resulted in your bariatric surgery operation being cancelled and/or delayed?
2. If you were in the process of the preoperative diet are you concerned within regaining the weight you have lost?
3. How has having your operation date cancelled and/or delayed made you feel?

**Post-Surgery (Display Logic with initial demographic questions)**

1. Since the COVID-19 outbreak, has your access to your vitamin and minerals supplements changed? Yes, No
   - If yes, please explain how your vitamin and minerals supplements have changed and any concerns you might have? (please specify in the text box)
   - Before the COVID-19 outbreak did you have access to a patient support group at your service? Yes No
   - If yes, what has happened to this support group? And if it has been cancelled how has that made you feel?

**Standard questions for all stages of the pathway**

1. How has the reduction in clinical service (i.e. access to your dietitian, doctor, surgeon) impacted on you? (please specify in the text box)
2. Has been enough support or information from your weight management or bariatric service since the COVID-19 outbreak? Yes No
   - Why do you believe you **have** received enough information? (please specify in the text box)
   - Why do you believe you **have not** received enough information and what more do you want to know? (please specify in the text box)
3. Since the COVID-19 outbreak, has the amount or type of communication you have had with your clinical service changed? Yes No
   - Describe how and why your communication with your clinical service has changed (please specify in the text box)
4. Are you aware of any guidance relating to people with obesity and COVID-19? yes no, if yes please stay which guidance
   - What are your thoughts of the guidance that has been given for people with obesity during the COVID-19 outbreak compared with other long-term health conditions such as diabetes? (In your answer please specify which guidance you are comparing to)

**Block 4: Impact on mental health, dietary and physical activity**

1. Since the COVID-19 outbreak, my mental health (i.e. mood, depression, anxiety) has been – much worse, worse, neither, better, much better

**Validated Questionnaires –** PHQ-9, Warwick Edinburgh

1. Has the way you shop changed since the COVID-19 outbreak? A great deal, a lot, a moderate amount, a little, not at all
   - Why and how has your shopping changed since the COVID-19 outbreak?
2. Has your diet changed since the COVID-19 outbreak? A great deal, a lot, a moderate amount, a little, not at all
   - Why and how has your diet changed since the COVID-19 outbreak?
3. Have you used food to manage your emotions during the COVID-19 outbreak? Yes No
   - Please can you explain has COVID-19 has resulted in you using food to cope emotionally? (please identify 3-5 key words)
4. Has your physical activity changed since the COVID-19 outbreak? A great deal, a lot, a moderate amount, a little, not at all
   - Why and how has your physical activity changed since the COVID-19 outbreak?
5. Has your sleep quality or quantity changed since the COVID-19 outbreak?
   - Approximately how many hours of sleep are you getting since the COVID-19 outbreak?
   - Why and how has your sleep changed since the COVID-19 outbreak?

**Block 5: Discrimination & Stigma**

1. Have you felt stigmatised or discriminated against since the COVID-19 outbreak due to your higher body weight? Yes, No, No, I felt stigmatised before the COVID-19 outbreak, No, I feel less discriminated against since COVID-19 outbreak
2. The UK Government guideline on social distancing describe people as “being seriously overweight”, what is your opinion on the use of this language? (please specify in the text box)
3. Please comment on anything else that you feel is important related to your care or service since the COVID-19 outbreak that has not been covered in this survey (please specify in the text box)

Thank you very much for completing this survey.

**Supplementary S2:** Response rate of the free text questions

| **List of the free text questions** | **Response rate (%)** |
| --- | --- |
| What is your biggest concern during the COVID-19? | 79.4 |
| The UK government has identified people who have a BMI 40kg/m^2^ or over as being at a higher risk of severe illness from COVID-19. Please describe how this made you feel? | 55.4 |
| How has following the UK Government decision for social distancing, lockdown, self-isolation and shielding made you feel? (please identify 3-5 key words) | 84 |
| How has having your appointments cancelled and/or delayed made you feel? (Tier 2) | 6.8 |
| How has having your appointments cancelled and/or delayed made you feel? (Tier 3) | 10.3 |
| How has having your initial appointment with the bariatric surgery team to discuss surgery cancelled and/or delayed made your feel? (Tier 4) | 7.4 |
| How has having your operation date cancelled and/or delayed made you feel? | 6.1 |
| Please explain how your access to your vitamin and mineral supplements have changed and any concerns you might have? | 3.9 |
| What has happened to this support group? And if it has been cancelled how has that made you feel? | 15.7 |
| How has the reduction in clinical service (i.e. access to your dietitian, doctor, surgeon) impacted on you? | 19.5 |
| Why do you believe you have received/not received enough information? | 23 |
| Describe how and why your communication with your clinical service has changed. | 18 |
| Are you aware of any guidance relating to people with obesity and COVID-19? | 14.7 |
| Why and how has your shopping changed since the COVID-19 outbreak? | 73.3 |
| Why and how has your diet changed since the COVID-19 outbreak? | 70.3 |
| Please can you explain how COVID-19 has resulted in you using food to cope emotionally? | 54.5 |
| Why and how has your physical activity changed since the COVID-19 outbreak? | 70.5 |
| Why and how has your sleep changed since the COVID-19 outbreak? | 52.3 |
| Have you felt stigmatised or discriminated against since the COVID-19 outbreak due to your higher body weight? | 45.9 |
| The UK Government guideline on social distancing describe people as being seriously overweight, what is your opinion on the use of this language? | 69.8 |
| Please comment on anything else that you feel is important related to your care or service since the COVID-19 outbreak that has not been covered in this survey. | 30.4 |

**Supplementary S3**: Examples of quotations for themes and subthemes

| **Themes** | **Sub-themes** | **Examples** |
| --- | --- | --- |
| Increased fear and anxiety | Fear of contracting and death from COVID-19 | *“Fear of death from contracting the virus and getting severe symptoms, needing hospitalisation, and dying alone”* (Participant 234 [P234]).  *“Catching COVID-19 and being very ill because I have type 1 diabetes, high blood pressure and obesity”* (P293).  *“I am concerned that should I contract COVID-19 that I will die or suffer long term poor physical consequences”* (P500).  *“I live alone. There is no one to help me if I become too ill. If make it to hospital, my size will be an issue for treatment”* (P60).  *“Should I get the virus, as a person living with obesity, I will not be treated. I won't be given chances that a slim person would”* (P66).  *“Dying and leaving my son without a mother”* (P239).  *“Dying - as I am a carer and a parent - who would look after my family”* (P460).  *“That I am more likely to die, that I bring it home to my family, who have respiratory issues, when out shopping”* (P278).  *“Getting the virus and bringing home to my 82 years old husband”* (P434). |
|  | Fear of weight gain | *“My family catching it and my weight gaining due to lack of available healthy foods and exercise”* (P135).  *“Gaining more weight and of course catching COVID”* (P521).  *“I'm scare of putting on more weight and being more risk. I'm trying to lose weight, monitoring calories, trying to go for a walk more”* (P278).  *“That I put on even more weight and I can’t return to work due to this as I struggled with work before COVID-19”* (P280).  *“Resorting to old eating habits due to anxiety and putting in weight. Concern that I won’t be approved for surgery”* (P257). |
| The impact of obesity being classified as ‘high risk’ | Heightened feeling of public shame and weight stigma | *“Vulnerable and ashamed that I may be a burden because of a condition that others perceive as preventable/my own fault”* (P239).  *“I worry that I won't receive the same level of care as other people if I do develop symptoms or have to go into hospital. It is my assumption that I will be judged for being fat and will be treated poorly”* (P43).  *“I feel like the ‘at greater risk’ statement implies if I get ill, I'm taking a bed from a more deserving person, that if I get ill, I don't deserve treatment because it is my own fault for being fat and lazy”* (P396).  *“Some of the comments online are disgraceful. Almost saying some people deserve to die because it’s their own fault”* (P12).  *“I feel demonised yet again. I have seen some very nasty posts on the Internet saying that if people are more vulnerable to COVID-19 because they are living with obesity, it is their own fault”.* (P253).  *“I first heard this info through my employer and was left feeling embarrassed and scared. There has not been enough access to information about the higher risk from the government and so I feel a lack of support”* (276).  *“Being 'seriously overweight' means we are in the vulnerable category, but the government have offered us no additional help”* (P326).  *“Worried because of it [government announcement linking obesity with severe COVID-19]. Determined to do something about my weight”* (P222).  *“I've been actively calorie counting to lose weight. I started doing this because I got scared hearing about how being 'seriously overweight' might make you more likely to get really sick from COVID-19”* (P544). |
|  | Response to the UK Government’s use of ‘seriously overweight’ to describe people living with obesity | *“It is confusing and fails to give a clear message. A few pounds could be seen as 'seriously overweight' by some. The lack of clarity is dangerous, and it sounds and feels discriminatory”* (P315).  *“I feel it is too vague and does not say what BMI would be considered 'seriously overweight'. Do they mean people with a BMI over 30 or 40?”* (P68).  *“It probably means different things to different people. It is too general. They need to be more specific such as give a BMI number that you would be between to be considered 'seriously overweight' or use the term obesity and explain that you are obesity if you have a BMI of over 35”* (P112).  *“Difficult to define what that is in layman's terms and open to interpretation”* (P55).  *“I find it offensive and demoralising”* (P17).  *“It's a bit harsh and could stigmatise overweight people”* (P310).  *“Serious is better than morbidly obese!”* (P48).  *“It's fine! Clear description! Grossly obese was worse!”* (P171).  *“I am 'seriously overweight' not sure what else they could say as not everyone knows their BMI”* (P83).  *“I think 'people living with overweight or obesity' is enough and gets message across. Morbidly and grossly obese have been used too and personally I find them offensive. Who on earth wants to be described as gross?”* (P295).  *“Yes, that's fine. It seems appropriate. It does not feel stigmatising”* (P391). |
| Disruption in weight management services | Cancellation of services due to UK Government restriction | *“Frustrated [of the Tier 2 service cancellation] as they could have been telephone appointments”* (P51).  *“I understand that we are in pandemic and other patients are higher emergency than I”* (P11).  *“I have been fine, but we haven't received any support. I understand completely though as hospital staff need to be diverted”* (P320).  *“No clinics running and no support groups. Impossible to get blood tests done too”* (P17).  *“Difficulty in obtaining B12 injections as I feel GPs do not understand the problems we have with malabsorption post-op”* (P172).  *“Very upset. I have attended weight management service for several years. I was finally ready to go for surgery and now I have to wait even longer. I’m worried I’ll gain weight as I’m in lockdown and then they will refuse my surgery”* (P455).  *“It’s made me lose track a little bit with my weight loss due to at the time of my one year face-to-face being cancelled, I was almost at my target for being put forward for weight loss surgery”* (P209).  *“Simply not enough information or support - we are where we are, and obesity is not a quick fix - I feel forgotten - it’s up to me to sort it for myself”* (P267). |
|  | Lack of communication between healthcare provider and patients | *“No one contacted me. No updates, no emails, or calls. Totally abandoned by my team”* (P246).  *“Reduction of services available. I was due to have an appointment for consideration of revision surgery which has been cancelled. I’ve had no contact from the hospital regarding where this leaves me”* (P276).    *“Had very little communication at all which is extremely disappointing”* (P472).  *“I haven’t heard from anyone so I don’t know if I can contact them. The unit was shut for a time, but I have no idea what’s going on. Left in limbo”* (P196).  *“Feeling lost and unaware of when next appointment will be, and hoping haven’t been lost in transition to next stage of my journey”* (P210).  *“I’ve tried to contact the team just for an update as I have not received anything not even a letter to say my operation will be delayed so it has made me feel very sad”* (P242).  *“I have received no real direct information from the service. It would be nice to have updates on whether things are being changed and perhaps on how we can maintain things from home”* (P189). |
|  | The importance use of technology to replace in-person support | *“I've not had to wait very long for a telephone appointment, had weekly Zoom meetings with Tier 2. Easier than having to go to certain venues for appointments”* (P282).  *“It hasn't been reduced. I consult with my dietitian regularly via video-calling”* (P367).  *“Not at all - everything is now done by phone/online”* (P395).  *“Maybe some online group sessions on Zoom or Skype etc. would have been helpful”* (P196).  *“I have had no contact with any service, online support would have been helpful”* (P83).  *“After the lockdown was nationally announced we were told that we will be monitored from a distance and received regular information via mail but as far as I am concerned, I received one information by mail only and then was left by myself without knowing what to expect. I was expecting a follow up or a new distance/ Zoom service”* (P404). |
| The impact on health-related behaviours | Consequences on dietary behaviour | *“Go to the shops less, write a list - don’t browse, try and plan meals more”* (P184).  *“I have shopped mainly online, and I have shopped quickly I don’t browse”* (P103).  *“Less frequently, online where able, alone when going to a shop as opposed to with partner, less availability of things I use”* (P40).  *“Buying more unhealthy snacks and alcohol”* (P68).  *“More ready meals - less fruit and vegetables as not able to get consistent delivery”* (P262).  *“Lack of fresh food and protein available. Also due to being unable to leave the house, snacking is happening. I graze a lot”* (P17).  *“When lockdown started there was hardly any meat and foods that I really needed and as I started to have to eat carbs again, it was a trigger for my bingeing. I started bingeing badly. I'm trying so hard not to. My mood dictates so much”* (P25).  *“Stress eating, more snacks, working from home means a change in routine. More alcohol intake and a lot more junk. I'm finding eating well more difficult. I've gained around a stone since lockdown”* (P88).  *“Overeating, eating more sweet things (biscuits and chocolate), grazing due to boredom and isolation”* (P104).  *“Fallen back on old patterns of comfort eating and binging to deal with anxiety and stress”* (P161).  *“Much more reliant on home cooking and much less on takeaways or ready meals”* (P155).  *“Making more effort to cook from fresh and avoiding processed foods”* (P302).  *“I now have to do far more cooking from scratch, which means more time and effort has to go into planning and preparing meals, washing up and getting the right ingredients into the house”* (P390).  *“Eaten less takeaways, less junk food. Working from home has made it easier for me to follow a low-carb, high-fat and intermittent fasting diet without the temptations of cakes in the office, and family getting takeaways”* (P20).  *“I have recently embraced a very low carb Mediterranean type of diet”* (P223).  *“I have been doing the total dietary replacement phase of 'counterweight'. Lockdown has made it much easier. No social pressure to eat”* (P364). |
|  | Consequences on physical activity | *“The gyms being closed so I cannot maintain my usual fitness routine”* (P377).  *“Before COVID I went to aquafit and swimming twice a week. I walked at least 30 mins most days. That's changed because swimming pools are closed and I'm terrified to leave the house to walk and most of the time just can't be bothered”* (P502).  *“Not able to attend swimming sessions or Tai Chi group. Isolated so not able to go for daily walk”* (P210).  *“Normally I go to the gym three times a week but now I can't. The stress has caused a rheumatoid arthritis flare which has hindered any activity at home”* (P43).  *“Working from home and so very little outdoor activity as have bad leg and find walking difficult”* (P162).  *“I have a very physical job which I haven't been doing and I can't exercise due to shielding”* (P224).  *“I have more time to exercise and should make more effort but motivating myself is harder at the moment”* (P104).  *“Gone from no exercise to walking 3 miles a day every day”* (P22).  *“I've been doing Joe Wicks every day which is a solid 20 minutes of HITT workout, something I never did before”* (P155)  *“I got time to have a hobby now. Where before I would lose 12 hours on a day to travelling and working at the office. I now save at least 4 hours a day which I now spend on gardening, exercising at home and my housework is done every day now instead of it building up for a few days and then having to do housework on weekends”* (P342).  *“I started the lockdown with a view to having far more time at home to work out. This worked well for the first 6-7 weeks, but I have found it very hard to motivate myself over the past few weeks and know that I am not working anywhere near as hard as I should, in order to maintain the weight loss”* (P382).  *“I actually started taking a weekly walk to get me out the house which I never did before and playing Just Dance. Although I have slipped back into not exercising at all”* (P528). |
|  | Consequences on sleep pattern | *“I sleep for maybe 3 hours at a time and then fall asleep lots throughout the day, I also suffer from chronic fatigue syndrome which I have struggled to manage without my daily active routine”* (P5).  *“Much harder to get to sleep and waking earlier, probably because I have a constant niggling feeling of anxiety”* (P43).  *“I never sleep through the night anymore but wake at least every 2-3 hours. I am napping during the day to make up for lost sleep. I feel sluggish and exhausted all the time”* (P391).  *“Because I was less active at first and worried about going out, I wasn’t getting much exercise, so I wasn’t sleeping great”* (P447).  *“Vivid dreams and feeling constantly tired”* (P141).  *“Improved. Not commuting and not getting stressed at work means I no longer wake in the night as much. Increased exercise probably helps too”* (P14).  *“Now getting more sleep! Getting 2 more hours sleep a night as no need to commute or get into work early. I am working from home”* (P250). |
| The adverse impact on mental health | *“So now with the Government warning feel more embarrassed and ashamed than ever which is having a huge impact on my mental health”* (P87).  *“Feeling low that my planned bariatric surgery was cancelled with less than a week to go because of the virus”* (P231).  *“Mental health and being away from family and friends”* (P511).  *“Resorting to old eating habits due to anxiety and putting in weight. Concern that I won’t be approved for surgery”* (P257).  *“My mental health has plummeted, because I'd waited a long time for counselling and had just started sessions, and the mental well-being activities I had started was in groups, so that stopped too”* (P393).  *“Mental low and I resort to old bad habits and default on my own”* (P160).  *“Due to feeling increased anxiety, low mood and social isolation I have experienced a relapse in binge eating disorder behaviours and weight has increased by 10 lbs since mid-March”* (P294).  *“I struggle to fall asleep some nights through stress and anxiety about COVID and what the future is going to be like, stress that I shouldn’t have put on this much weight etc.”* (P280).  *“I couldn't get any mental health support at all, and poor mental health leads to being unable to maintain a regular diet. It kicks off bingeing and starving”* (P394).  *“I feel people are being left and people’s health is suffering and that will cause far worse issues long term than pandemic has. I worry for the future like it stands, and impact on mental health specifically”* (P352).  *“Mental health and obesity often go together, I think they should be tackled together in a better way rather than relying on fixing one to sort the other”* (P311). | |
